# Bed capacities and disinfection practices in hospitals in Istanbul are correlated

**DOI:** 10.1186/s12941-015-0073-5

**Published:** 2015-03-19

**Authors:** Bahri Teker, Aziz Ogutlu, Mesut Yilmaz, Serap Gencer, Oguz Karabay

**Affiliations:** Department of Clinical Microbiology and Infectious Diseases, Private Nisa Hospital, Istanbul, Turkey; Department of Clinical Microbiology and Infectious Diseases, Medicine Faculty of Sakarya University, Sakarya, Turkey; Department of Clinical Microbiology and Infectious Diseases, Medicine Faculty of Medipol University, Istanbul, Turkey; Department of Clinical Microbiology and Infectious Diseases, Kartal Dr. Lutfi Kirdar Education and Research Hospital, Istanbul, Turkey; Health Science Institute of Sakarya University, Sakarya, Turkey

**Keywords:** Sterilization, Disinfection, Antisepsis, Disinfection error, Private hospital

## Abstract

**Background:**

Disinfection, antisepsis and sterilization (DAS) practices are of critical importance in hospital practice. This study aims to investigate the daily DAS practices of private hospitals in Istanbul, Turkey.

**Methods:**

The DAS practices of 155 private hospitals in Istanbul province were investigated using a questionnaire including 26 questions. The questionnaire forms were faxed to all private hospitals located in Istanbul. A *p* value < 0.05 accepted as significant.

**Results:**

The 75 [48%] hospitals out of 155 hospitals responded. The quality of DAS practice was correlated with hospital bed capacity. In these hospitals, glutaraldehyde (27%) was the most common chemical used to disinfect endoscopy instruments. The rate of availability of air gun in endoscopy units in these hospitals was significantly associated with hospital bed capacity (p <0.001). Sticky mats placed at doors of risky areas were not reported to be used in the large bed capacity (LBC) hospitals unlike the small bed capacity (SBC) hospitals where 50% of these hospitals reported to use the sticky door mats (p =0.0144).

**Conclusions:**

Private hospitals in Istanbul need in-service training towards sterilization and disinfection issues. It is concluded that private hospitals need policies and educational activities for DAS practices.

**Electronic supplementary material:**

The online version of this article (doi:10.1186/s12941-015-0073-5) contains supplementary material, which is available to authorized users.

## Introduction

Most microorganisms capable of nosocomial infections can survive for weeks or even months on patients, healthcare providers, and in the hospital environments [1]. These microorganisms can then spread to other patients from these sources [2].

Most of nosocomial infections can be prevented if the standard infection control precautions are rigorously applied. Sterilization units are of critical importance for sterilization procedures in hospitals and are required to serve in accordance with the established standards. Indeed, achieving and maintaining the required standards is essential for quality of health care since incomplete sterilization and untrained staff can cause nosocomial infections [3].

The standards implemented in the Turkish public and university hospitals are consistent with the minimum disinfection, antisepsis and sterilization [DAS] requirements accepted worldwide [4]. However, we could not achieve any data indicating to what extent the DAS standards are being implemented in the private hospitals serving thousands of people every day in Istanbul where approximately 14 million people live. With this study, investigation of some of the key indicators regarding DAS practices were aimed for the first time reaching 155 private hospital serving in Istanbul.

## Materials and methods

### Survey

A survey consisting of 26 multiple-choice questions and investigating the hospitals’ DAS practice was prepared for each private hospital (Additional file “1”, questionnaire form). This survey has been sent to managing directors of all the private hospitals through the Istanbul Provincial Directorate of Health. People who were identified by the Infection Control Committees in the private hospitals were asked to respond to the survey questions.

### Following guideline

We compared the hospitals practices with national DAS guideline [5].

### Hospitals

There were 155 private hospitals in Istanbul at the date of this study. 56 private (36.1%) hospitals answered the survey in the first place. Upon sending a reminder to the hospitals which did not respond, 19 (12.3%) more hospitals answered and responded to the survey. As a result, the results of the 75 hospitals (48.4%) responded were evaluated.

### Statistical analysis

The data was recorded in MS Excel format. The data were analyzed using EPI Info ver. 6.0 [CDC, Atlanta-USA] program. Chi-square test and Fisher’s exact test were used for statistical significance. P < 0.05 was considered to be significant.

## Results

Seventy five (48.4%) of 155 hospitals responded to the survey. When bed capacities of the 75 hospital responding to this study are evaluated, it is seen that 26 hospitals (34.7%) have a capacity under 50 beds (small capacity, SC) and 41 hospitals (54.7%) have a capacity of 50 beds and above (Large capacity, LC). No information about the bed capacity of 8 hospitals (10.7%) was available. It was observed that the hospitals with LC used alternative methods in some implementations of DAS practices (Table 1).

**Table 1:** **Comparing the hospitals based on their bed capacities**

**Parameter**	**Large-scale hospital >50**		**Small-scale hospital <50**		Total*		**P value**
	**n**	**%**	**n**	**%**	**N**	**%**	**p**
Overshoes are worn when entering into intensive care unit	22	54	19	73	43	57	0.0617
Sticky mats are used	8	20	13	50	24	32	0.0144
Gas-plasma system is available	9	22	1	4	12	16	0.0780
Central sterilization unit (CSU) is available	34	83	16	62	57	76	0.0823
A physician responsible for CSU is available	15	37	8	31	25	33	0.5958
Sterilization records can be accessed retrospectively	39	95	25	96	72	96	1.0000
Air gun is used in washing unit	31	76	8	31	45	60	0.0003
Ethylene oxide sterilizer is available	13	32	40	15	18	24	0.1555
Automatized endoscope washer in operating room is available	19	46	7	27	30	40	0.1245
Endoscopy unit is available in hospital	39	95	17	65	60	80	0.0022
Automatized endoscope washer is available in endoscopy unit	20	49	8	31	31	41	0.5914
At least one of the autoclaves is older than 10 years	13	32	0	0	14	19	0.0009
Ground disinfectant is used in inpatient services	40	98	24	92	72	96	0.5550

*No data about the bed capacity of 8 hospitals (10.7%) was available.

While 23 (31%) of the hospitals used alcohol-based antiseptics for hand hygiene alone, 11 of the hospitals (15%) used alcohol with other compounds methods. The remaining 34 hospitals (45%) used soap and alcohol-based hand antiseptics together.

The types of chemicals used to disinfect endoscopy instruments in these hospitals were as follows; 20 hospitals (27%) glutaraldehyde, 6 hospitals (8%) Ortho-phthalaldehyde [OPA], 22 hospitals (29%) peracetic acid, 6 hospitals (8%) hydrogen peroxide, 6 hospitals (8%) quaternary ammonium, 16 hospitals (21%) another (quaternaryammonium + biguanide, glutaraldehyde + phenol/phenate, H_2_O_2_ + peracetic asid) disinfectant.

The following chemicals were reportedly used to disinfect floors and surfaces of the intensive care units; 29 hospitals (39%) bleach or chlorine tablets, 3 hospitals (4%) phenol compound, 11 hospitals (15%) quaternary ammonium, 1 hospital (1%) solution containing formaldehyde, 24 hospitals (32%) didecyl dimethyl ammonium chloride, 22 hospitals (29%) mixtures of these compounds.

And the chemicals used to disinfect floors and surfaces of the operating rooms were; 23 hospitals (31%) bleach or chlorine tablets, 3 hospitals (4%) phenol compound, 11 hospitals (15%) quaternary ammonium, 2 hospitals (3%) solutions containing formaldehyde, 23 hospitals (31%) didecyl dimethyl ammonium chloride, 25 hospitals (33%) mixtures of these compounds.

72 hospitals (96%) reported that floors were disinfected in inpatient services. The reported types of disinfectant materials used by the hospitals for this purpose were as follows; 48 hospitals (64%) bleach or chlorine tablets, 3 hospitals (4%) phenol compound, 11 hospitals (15%) quaternary ammonium, 1 hospital (1%) solutions containing formaldehyde, 18 hospitals (24%) didecyl dimethyl ammonium chloride, 20 hospitals (27%) mixtures of these compounds.

## Discussion

In this study we examined disinfection and sterilization practices of 75 private hospitals in Istanbul. Interestingly, we observed that the most widely used disinfectant for endoscopy instruments was glutaraldehyde (27%), which may be attributed to economic reasons. OPA is 7 to 10 times more expensive than gluteraldehyde. It is essential that environments where glutaraldehyde is applied should be well ventilated because of its potentially toxic vapor to human body. Use of this product by hospitals without adequate ventilation threatens the staff health. Another reason why this chemical is widely used may be due to the fact that it has been used in hospitals for ages and well known by health care providers. If private hospital administrators are trained about existence and advantages of alternative products, preferences of private hospitals for endoscopy disinfection may change. Also, the endoscope manufacturers recommend use of glutaraldehyde as disinfectant among others [6,7].

The most widely used chemical in high risk contamination areas, such as operating rooms and intensive care units, was bleach (39%). This is the practice proposed in many countries and a relatively a cheaper alternative. Remarkably, most of the hospitals responded to the survey (61%) do not use bleach. This may be due to the effect of bleach resulting smell and headaches [8,9].

The use of quaternary ammonium (15%) in the intensive care units is thought provoking. It should be noted that quaternary ammonium compounds are bacteriostatic and are ineffective to Hepatitis B and *Pseudomonas aeruginosa* [10].

Although it is enough to clean the inpatient services of the hospitals just with water and detergent –except high risk areas and fomites (surfaces frequently touched by hands such as door handles, bed-heads and bedside cabinets, tap handles etc.), the hospitals use 4% phenol compound, 14% quaternary ammonium chloride, 24% didecyl dimethyl ammonium chloride. This may be due to a lack of knowledge on the DAS practice [11].

When the use of sticky door mats in the intensive care units was examined, it was observed that private hospitals with small-bed capacity (<50 beds) significantly preferred this method. However, the use of sticky doormats before entering into the high risk areas is not recommended except for controlling dust [12]. The fact that the average rate for wearing overshoes when entering into the intensive care units is 57% indicates that the guidelines are not adequately observed [12]. This may be due to employment of an Infectious Diseases and Clinical Microbiology Specialist by the hospitals and presence of effective Infection Control Committee established by these hospitals. In addition, we think that this situation may arise when the training for infection control in the hospitals are insufficient.

The use of air gun in the washing units was found to be significantly higher in the LBC hospitals (p = 0.0003). The fact that SBC hospitals do not allocate additional resources for washing guns may be due to financial concerns. 

The correct sterilization technics of health procedures, invasive implements, materials and equipment used in direct patient care and surgery directly influences patient care. Our study showed that some may be some overlaps. To prevent this purpose it is important that controls raising the required too. These controls should be in the form of both internal and external control. External control official authority should be involved.

## Conclusion

We conclude that private hospitals in Istanbul need in-service training DAS issues. It is also concluded that private hospitals in Istanbul need policies that will standardize the DAS practices and DAS training.

## Additional file

Additional file 1: Questionnaire.
