# Supplementary material for: Bed capacities and disinfection practices in hospitals in Istanbul are correlated
Source: Ann Clin Microbiol Antimicrob. 2015 Mar 19;14:13. doi: 10.1186/s12941-015-0073-5 (PMC4458342; doi:10.1186/s12941-015-0073-5)
Supplement: Additional file 1: — Questionnaire. [file 12941_2015_73_MOESM1_ESM.doc]

# **QUESTIONNAIRE**

Number: Date:

Bed capacity:

**Questions**

1. Which of the following hand hygiene practices used in the service?

a) Rigid soap b)liquit soap c)Medical soap d) Alcohol-based hand rubs e) Others (………….)

2. Does overshoes worn when entering the intensive care?

a)Yes b)No

3. Operating room, Do you use the sticky mat any of entries in the operating room, intensive care unit, etc.

a)Yes b)No

4. Does your hospital have a gas-plasma system (H2O2, formaldehyde, etc.)

a)Yes b)No

5. What is done with the control of the gas-plasma system?

…………………………………………………………………………..

6. What products do you use the endoscope disinfection?

a) glutaraldehyde b) ortho-phthalaldehyde c) peracetic acid

d) Hydrogen peroxide e) Quaternary ammonium f)Others (……….)

7. If you are using glutaraldehyde, what do you do with the check?

…………………………………………………………………………….

8. Where do you do the glutaraldehyde disinfection process?

……………………………………………………………………………

9. How often do you use a biological indicator?

a) Once a day b) More than once a day c) Once a week

d) Once a month e) Don’t use biologic indicator

10. When do you get the results of biological indicators?

a) < 2 hours b)2-6 hours c) 6-12 hours d) 12-24 hours e)> 24 hours

11. How often do you use Bowie-Dick test?

a)Everyday, first use b)Every use c) Every 3 days d)Once a week e)Don’t use

12. Is there a central sterilization unit (CSU)?

a)Yes b)No

13. Do you have a physician responsible for the MS?

a)Yes b)No

14. How often training the CSU staff?

a)Once a week b)Once a two weeks c)Once a month

d) Every 6 months e)Once a year f)Others (……………)

15. Can you reach your sterilization records retrospectively?

a)Yes b)No

16. How does the sterilization record?

a) Writing to the registry sterilization b) Writing in the patient file

c)a+b both d) Records are kept electronically e)No records f)Others (…………)

17. Is there air gun in the washing unit?

a)Yes b)No

18. How old you are using an autoclave? (If you have more than one, please write each of them separately)

a)< 5 years b)5-10 years c)> 10 years

19. Which of the following disinfectant do you use in the inpatient services?

a)Bleach b) Chlorine tablets c) Phenolic compound d) Quaternary ammonium

e) Solutions containing formaldehyde e) Didecyl dimethyl ammonium chloride f) Others (………)

20. Which of the following disinfectant do you use to place and surface in intensive care?

a)Bleach b) Chlorine tablets c) Phenolic compound d) Quaternary ammonium

e) Solutions containing formaldehyde e) Didecyl dimethyl ammonium chloride f) Others (…………)

21. Which of the following disinfectant do you use to place and surface in operating room?

a)Bleach b) Chlorine tablets c) Phenolic compound d) Quaternary ammonium

e) Solutions containing formaldehyde e) Didecyl dimethyl ammonium chloride f) Others (…………)

22. Do you have ethylene oxide?

a)Yes b)No

23. Is there an automatized endoscope washer in the operating room?

a)Yes b)No

24. Does endoscopy (gastroscopy, rectoscopy, bronchoscopy, etc.) done?

a)Yes b)No

25. Is there an automatized endoscope washer in the endoscopy unit?

a)Yes b)No

26. What is the most commonly isolated bacteria in your ICU?

a)Methicillin-resistant Staphylococcus aureus b)Enterococcus spp. c)Acinetobacter spp.

d)Pseudomonas aeruginosa e)Escherichia coli e)Others (……….)
